# Mycosis Is a Disease State Encountered Rarely in Shore Crabs, *Carcinus maenas*

**DOI:** 10.3390/pathogens9060462

**Published:** 2020-06-11

**Authors:** Charlotte E. Davies, Sophie H. Malkin, Jessica E. Thomas, Frederico M. Batista, Andrew F. Rowley, Christopher J. Coates

**Affiliations:** 1 Department of Biosciences, College of Science, Swansea University, Swansea SA2 8PP, Wales, UK; s.h.malkin@swansea.ac.uk (S.H.M.); jessica.e.thomas@swansea.ac.uk (J.E.T.); frederico.batista@cefas.co.uk (F.M.B.); 2 Centre for Environment, Fisheries and Aquaculture Science (Cefas), Barrack Road, The Nothe, Weymouth DT4 8UB, UK

**Keywords:** marine fungi, phylogeny, histopathology, parasite, disease connectivity, fisheries

## Abstract

There is a paucity of knowledge regarding the diversity and impact(s) of disease-causing fungi in marine animals, especially shellfish. In efforts to address this knowledge gap for the shore crab *Carcinus maenas*, a year-long disease screen was carried out across two sites in Swansea Bay (Wales, UK) with a view to characterising putative fungal infections. Crabs were sampled between November 2017 and October 2018, and screened systematically for disease signatures using haemolymph (blood) preparations, targeted PCR and tissue histopathology. Strikingly, mycosis was confirmed in ~0.4% of total crabs tested (*n* = 1191) and restricted to one location only (Mumbles Pier). Clinical infections were observed in four out of four infected crabs. In these animals, the gills and hepatopancreas were congested with fungal morphotypes. In addition, some evidence indicates haemocyte (immune cell) reactivity toward the fungi. Phylogenetic placement of the partial internal transcribed spacer (ITS1) gene regions amplified from three mycotic crabs revealed the causative agent to be related to hypocrealean fungi, thereby representing a novel species.

## 1. Introduction

Crustaceans are known to be infected by a number of disease-causing agents, including bacteria and viruses, however, infections caused by fungi and oomcyetes are reported rarely [1]. Compared to the terrestrial environment, much less is known about fungal diversity and ecology in aquatic habitats. Nevertheless, fungal infections have been observed in a range of marine environments and hosts from macroalgae, seagrass, mangrove, coral, and crustaceans to marine mammals [2]. A recent review estimated that the 1100 species of marine fungi identified may only represent ~10% of the total species present [3,4]. A well-documented pathogen of Crustacea is the causative agent of crayfish plague, *Aphanomyces astaci* [5], a pathogenic oomycete [6]. Previously, some oomycetes have been incorrectly described as fungi; however, although fungi and oomycetes both grow as filamentous hyphae, their lineages diverged before the split of fungi from plants and animals [7]. Other oomycetes, *Haliphthoros* sp. have also been reported in post larval spiny (*Jasus edwardsii*) and clawed (*Homarus gammarus, H. americanus*) lobsters in aquaculture facilities around the world, but never in the wild [8,9,10,11]. Recently, the first recorded occurrence of *Halioticida noduliformans,* a Haliphthoros-like parasitic oomycete thought to lead to reduced fecundity in *H. gammarus* was reported [12]. In the case of fungal infections of crustaceans, there are very few examples that have been extensively studied in terms of their virulence, pathobiology and host response.

*Carcinus maenas* is a crustacean species native to all coasts of the UK, Ireland and North-East Atlantic. It is well known for its role as a prolific invasive to many other parts of the world [13], as well as its capacity to harbour numerous parasites and diseases [14,15,16,17,18]. Of late, parasites of *C. maenas* are of increasing interest and importance due to their disease status. For example, this species has been found to host two novel viruses [17,19], to be a vector for ostreid herpesvirus-1 microvariant [15] and contain a range of other endo- (*Haplosporidium* sp. [20], *Hematodinium* sp. [14]) and ecto-parasites [16,18]. In addition, *C. maenas* is an ideal model species for diseases of allospecifics due to its versatile diet and habitat choice, meaning it shares environments with an abundance of economically important shellfish species, e.g., edible crabs (*Cancer pagurus*), European lobster (*Homarus gammarus*) and velvet swimming crabs (*Necora puber*) [21].

Upon examining *C. maenas* at the tissue (histopathology), cellular (haemolymph) and molecular (PCR) levels, we have gathered evidence of a rare, novel fungal infection. Shore crab mycosis was observed in a single site, Mumbles Pier in Swansea Bay (South Wales, UK), suggesting this disease is not endemic in this host.

## 2. Results and Discussion

### 2.1. General Population Observations

Overall, 1191 crabs were sampled across the year-long survey; 603 from the Dock and 588 from the Pier. Of these crabs, four individuals (~0.3%), all from the Pier, were seen to contain fungal infections via haemolymph preparations of live cells viewed using phase contrast microscopy. Following this, a subsample of 324 crabs was tested for fungus via PCR. Of these further 324 crabs analysed using PCR, one additional crab was positive for fungal presence at a subclinical level, again, from the Pier location. The affected crabs measured 40.6 ± 5.3 mm (mean, ±SD) in carapace width, were of both sexes, and appeared throughout the year with seemingly no discernible seasonal pattern (Table 1).

### 2.2. Haemolymph Preparations

**Figure 1:**
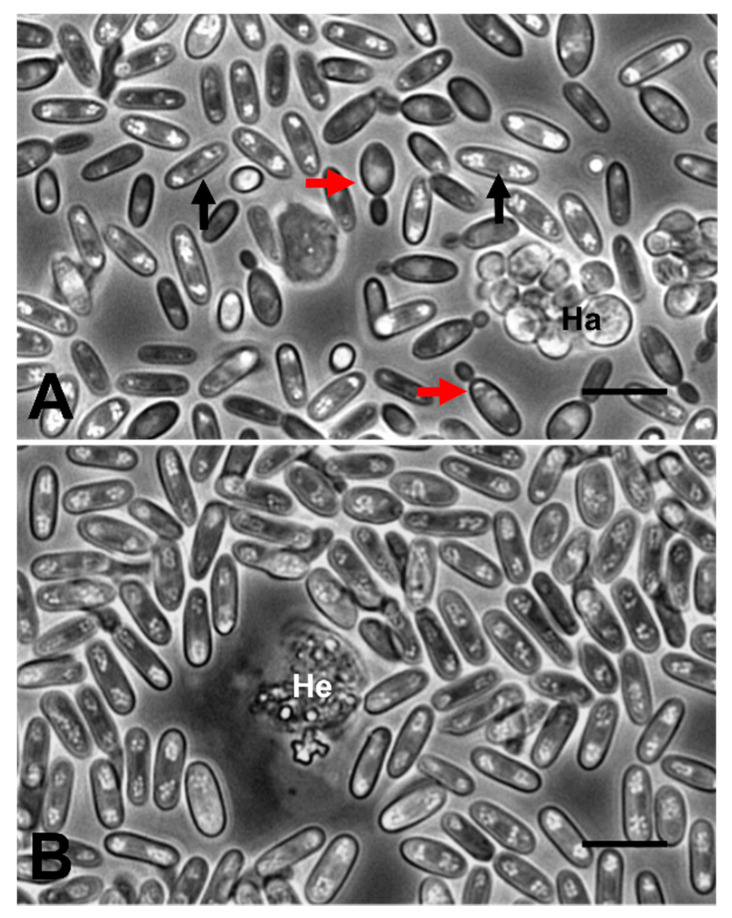
Phase contrast micrographs of haemolymph preparations from shore crabs, *C. maenas*, infected with the fungus. (**A**) Budding (red unlabelled arrows) and spore-like (black unlabelled arrows) forms of the fungus together with haplosporidians (Ha). (**B**) Spore-like forms of the fungus and a *Hematodinium* (He) trophont. Scale bars = 10 µm.

Crabs infected by the fungus had cloudy and viscous haemolymph. Observations of haemolymph preparations showed large numbers of fungal elements consisting of yeast- and spore-like forms (Figure 1A,B). These preparations had few circulating haemocytes. The haemolymph of 1 of 4 crabs also contained clumps of unicellular haplosporidians (Figure 1A). In addition, two more individuals were infected with the dinoflagellate parasite, *Hematodinium* sp. (Figure 1B), meaning 75% of animals infected with the fungus were co-infected. Interestingly, Stentiford et al. [22] and Smith et al. [23] also witnessed such a co-infection in *C. pagurus* and, although morphologically similar to the current infections, they were not the same species of fungus.

### 2.3. Histology

**Figure 2:**
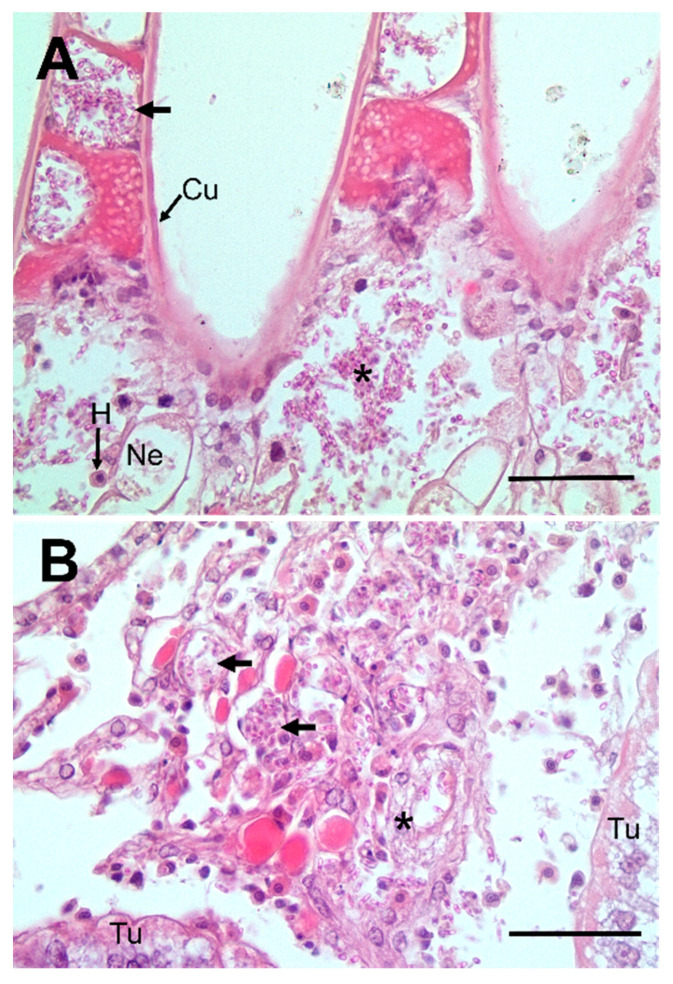
Histopathology of fungal infections in shore crab, *C. maenas*. (**A**) Section through the gill showing numerous free fungal elements in the stem (*) and lamellae (arrow) of the gill. Nephrocytes (Ne) in the gill stem are swollen. Note few circulating haemocytes (H) present. (**B**) Section through the hepatopancreas of a fungus-infected crab with large numbers of fungal elements in the haemolymph channels (unlabelled arrows). The fixed phagocytes that surround some channels are swollen but free of intracellular fungi (*). Hepatopancreatic tubule (Tu). Scale bars = 50 µm.

**Figure 3:**
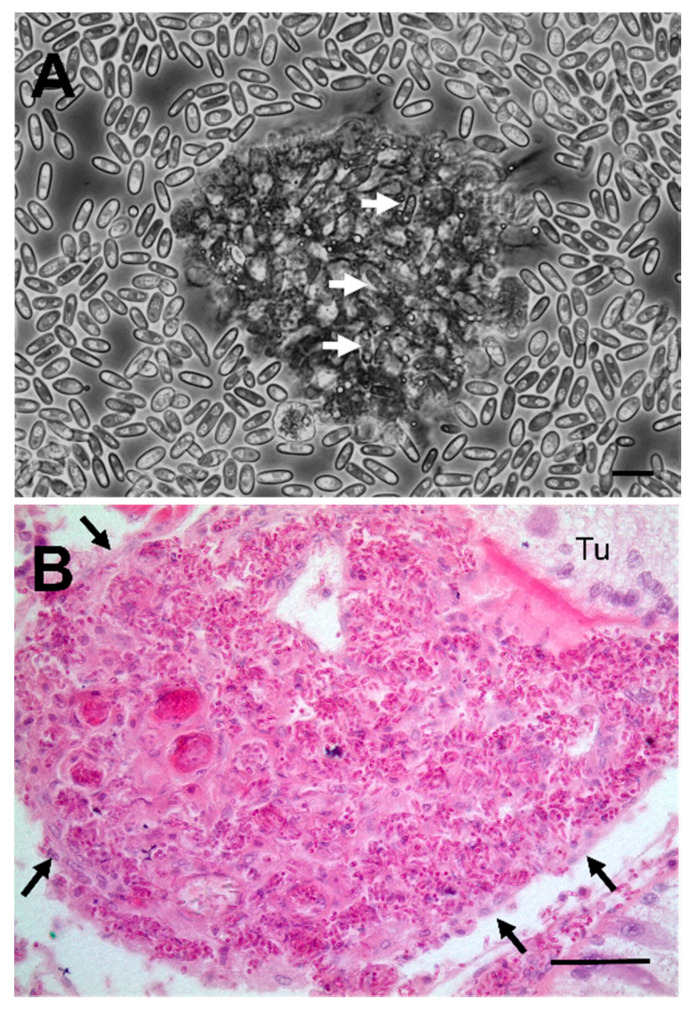
Interaction between the host’s immune system and the invading fungus. (**A**) Phase contrast micrograph of a clump of degenerating haemocytes with internalised fungi (unlabelled arrows). Scale bar = 10 µm. (**B**) Large clump of fungi and haemocytes in the intertubular space of the hepatopancreas. Note the thin wall of ensheathing haemocytes surrounding the clump (unlabelled arrows). Scale bar = 50 µm.

Histological examination of crab tissues showed the presence of numerous fungal elements in the haemal space in gill stems and lamellae (Figure 2A). The nephrocytes in the haemal space were swollen but did not contain any fungi (Figure 2A). Fungi were also found in the intertubular spaces of the hepatopancreas in haemolymph channels and free in the connective tissue (Figure 2B). The fixed phagocytes surrounding the haemolymph channels were swollen but free from intracellular fungi (Figure 2B). There was limited evidence of extensive immune reaction to the presence of fungi, both in the haemolymph and other tissues such as the gills and hepatopancreas. The haemolymph of infected crabs contained small clumps (termed nodules) of haemocytes containing seemingly intracellular fungi (Figure 3A). There were occasional loose clumps of haemocytes surrounding free fungi in the intertubular spaces in the hepatopancreas (Figure 3B). No clumps of haemocytes were seen in the gills (not shown).

The histological appearance and tissue distribution of fungi observed in the current study is similar to that reported in edible crabs (*Cancer pagurus*) [23]. In both cases, some of the crabs infected with fungi also had co-infections including the dinoflagellate parasite, *Hematodinium*, that is commonly found in both shore and edible crabs at this site [24]. Therefore, it is probable that fungi are rarely the primary infectious agent and rely on the presence of other parasites/pathogens to damage the host’s immune system, leaving them vulnerable to such secondary infections. Smith and Rowley [25] conducted a long-term infection trial with edible crabs artificially infected with *Hematodinium*. They found that of the crabs that became infected, nearly 50% developed secondary fungal infections that were similar to that previously described in field-collected crabs [23]. Death of the hosts appeared to be caused by the presence of both *Hematodinium* and the fungus. In the present study, the one crab infected with both the fungus and a *Haplosporidium* sp. (Figure 1A) also harboured encysted trematode parasites in the hepatopancreas (not shown).

There is some evidence for a localised haemocyte-response to fungi—depicted by haemocyte degeneration—but there is little evidence for a concerted host response to the presence of the fungus in all major tissues. Infected crabs had very few such cells in circulation and when seen they were in clumps containing internalised fungal elements. Although haemocyte counts were undertaken during the survey, for these infected animals, counts were not possible due to the haemolymph being overrun with fungal morphotypes (Figure 3A). In other tissues, the majority of fungi were free in circulation with occasional, partially formed nodules with a few flattened haemocytes apparently attempting to wall off these pathogens (Figure 3B). Such defence reactions are clearly inefficient as the fungi appear to overcome the host’s immune system, leading to systemic mycosis.

### 2.4. Phylogenetic Analyses

**Figure 4:**
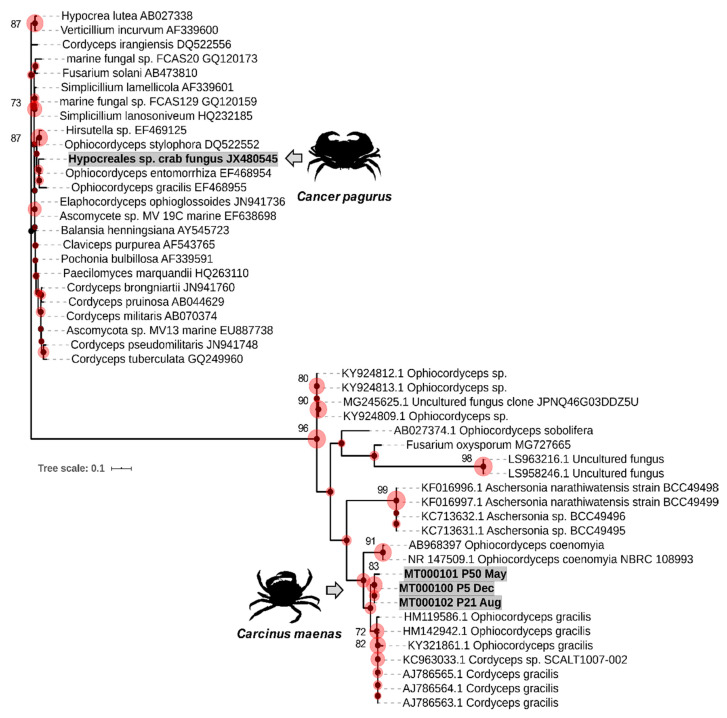
Consensus phylogram (unrooted) of the partial ITS1 gene region of sequences recovered from compromised shore crabs, *Carcinus maenas* (MT000100–MT000102). Reference sequences represent the top 20 BLASTn-search results from NCBI plus a comprehensive marine fungi alignment adapted from Smith et al. [22] Bootstrap support values are depicted as spheres (partitions >70 are included). The scale represents nucleotide substitutions per site (maximum likelihood estimation, 1000 bootstrap replicates). Inset; silhouettes of crab hosts, *C. maenas* and edible (brown) crab *Cancer pagurus,* are placed next to their pathogenic fungi (grey arrows).

Partial internal transcribed spacer (ITS1) sequences of the crabs positive for fungi by microscopical examination or group-specific PCR were aligned, showing clearly unique sequence types. Each crab harboured just one fungal ecotype/infection. These were BLAST-searched against the NCBI GenBank nucleotide database. The top match, which was also characterised, for all sequences was accession number HM119586 (87.88–88.55% identity, 100% coverage), corresponding to *Ophiocordyceps gracilis*, a fungus used in studies for Traditional Chinese Medicine reported in Zhong et al. [26] (now *Paraisaria gracilis* [27]). *Ophiocordyceps* species can exist in both hyphal and budding forms—the latter being similar to our observations (see Figure 1). Maximum likelihood phylogenetic analyses (Figure 4) revealed that all sequences branch within the paraphyletic assemblage of lineages that currently comprise the genera *Ophiocordyceps*, *Aschersonia* and *Fusarium.* The tree is comprised of fungi isolated from the marine environment (including a decapod), as well as known hypocrealean entomopathogens and plant pathogens. All three novel sequences were markedly different from the characterised lineages available. Notably, an *Ophiocordyceps*-like fungal infection of *C. pagurus* (*Hypocreales* sp. described by Smith et al. [23]) from the same Swansea Bay site (Mumbles Pier) was placed within a different clade away from the shore crab (*C. maenas*) sequences (Figure 4). Fewer than 10% of edible crabs were infected with the so-called *Hypocreales* sp. and usually they co-occurred with another parasite, namely *Hematodinium* sp. Overall, we posit that such fungal infections of decapods are rare—at least in this bay environment—and likely take hold in effete animals only.

## 3. Materials and Methods

### 3.1. Survey Sites and Sample Collection

Between November 2017 and October 2018, the populations of shore crabs, *Carcinus maenas,* were sampled at the Prince of Wales Dock, Swansea (51°37′8.76″ N, 3°55′36.84″ W) and Mumbles Pier (51°34′8.958″ N, 3°58′33.297″ W), both in South Wales, UK. Crabs were sampled once a month, for 12 months, using strings of baited traps, immersed for 24 h. Once retrieved, 50 crabs were chosen at random from the pots, individually bagged and transported on ice back to the laboratory for processing the same day. For full details, see Davies et al. [14], but briefly, crabs were measured (carapace width or CW; mm), sexed and other biometric parameters were noted (moult stage, fouling, pigment loss, shell disease, limb loss, carapace colour). In addition, ~350 μL of haemolymph was taken using a 23-gauge hypodermic needle fitted with a sterile 1-mL syringe. Haemolymph was separated, total haemocyte counts recorded, haemolymph preparations examined, and the remainder of haemolymph was stored at −80 °C for subsequent DNA processing.

### 3.2. DNA Extraction and Quantification

Genomic crab DNA was extracted from 100 µL of thawed haemolymph using Qiagen Blood and Tissue Kits (Qiagen, Hilden, Germany) following the manufacturer’s instructions. Extracted DNA was quantified using a Qubit^®^ dsDNA High Sensitivity Assay Kit and Qubit^®^ Fluorometer (Invitrogen, Carlsbad, CA, USA).

### 3.3. PCR and Sequencing Conditions

Fungi-specific PCR reactions were carried out using forward primer ITS1F CTTGGTCATTTAGAGGAAGTAA [28] and reverse primer ITS2 GCTGCGTTCTTCATCGATGC [29], producing amplicons of 320 bp using a protocol modified from [30]. PCR reactions were carried out in 25 μL total reaction volume using 2X Master Mix (New England Biolabs, Ipswich Massachusetts, MA, USA), 0.4 mM oligonucleotide primers synthesised by Eurofins (Ebersberg, Germany), 1 μL DNA (ca. 50–200 ng/μL) and performed on a BioRad T100 PCR thermal cycler. Cycling conditions were as follows: 95 °C for 2 min, followed by 30 cycles of 95 °C for 30 s, 55 °C for 30 s, 72 °C for 1 min and finalised by 10 min elongation at 72 °C. Products derived from PCR were visualised on a 2% agarose/TBE gel with GreenSafe premium nucleic acid stain (NZYTech, Portugal). Sanger sequencing was performed using both forward and reverse primers synthesised by Source BioScience (Nottingham, UK) and Eurofins. Sequences were deposited in GenBank under accession numbers MT000100-MT000103.

### 3.4. Histology

Tissue histology took place according to Davies et al. [14] and was used as the secondary tool after PCR, to estimate the severity of, and potential host immune responses to, any fungal infection. Briefly, gills and portions of the hepatopancreas/gonad were excised and fixed in Davidson’s seawater fixative for 24 h prior to their storage in 70% ethanol. Samples were dehydrated in a graded series of ethanol, transferred to Histoclear/Histochoice (Sigma-Aldrich, Dorset, UK) and infiltrated with molten wax using a Shandon™ automated tissue processor (Thermo Fisher Scientific, Altrincham, UK) prior to embedding. Blocks were cut at ca. 5–7 µm thickness using an RM2245 microtome (Leica, Wetzlar, Germany). Sections were mounted on glass slides using glycerine albumin and stained with Cole’s haematoxylin and eosin. Stained slides were viewed and imaged using an Olympus BX41 microscope. Images were adjusted for colour balance and contrast only.

### 3.5. Phylogenetic Analyses

The three new ITS1 sequences were searched against GenBank using the Basic Local Alignment Search Tool (BLASTn) and the top 20 closest matches were retrieved. These sequences were added to a further 26 marine fungi reference sequences to make up a comprehensive dataset. Sequences were trimmed manually, and aligned using the Clustal tool in MEGA X. Evolutionary analyses and reconstructions were carried out in MEGA X [31] using the maximum likelihood routine based on the Tamura-Nei model. A consensus tree with the highest log likelihood value (−1428.17) from 1000 bootstrap re-samplings was presented using iTOL software [32].

## 4. Conclusions

After surveying ~1200 shore crabs using diagnostic techniques across tissue (histopathology), cellular (haemolymph) and molecular (PCR) levels, we encountered four animals with clinical fungal infections. Phylogenetic placement of the PCR amplicons (partial ITS1 gene region) and appearance of budding morphotypes revealed the disease-causing agent to be a novel species related to *Ophiocordyceps gracilis*, but distinct from a fungal disease reported for edible crabs in the same location. Shore crab mycosis was observed in few animals in a single site, Mumbles Pier in Swansea Bay (South Wales, UK), suggesting this disorder is opportunistic rather than endemic.

## Figures and Tables

**Table 1:** Biometric and temporal data of crabs found with clinical mycosis.

	P5_Dec	P50_Dec	P50_May	P21_Aug
Date	December 2017	December 2017	May 2018	August 2018
Carapace size	42.9 mm	47.5 mm	33 mm	39 mm
Sex	Female	Male	Female	Female
Fouling *	Yes	No	No	No
Carapace colour	Yellow	Yellow	Yellow	Yellow
Limb loss	0	3	0	0
Haemolymph colour	Milky	Cloudy	Milky	Milky
Co-infection	*Hematodinium* sp.	Encysted trematodes; *Haplosporidium carcini*	N/A	*Hematodinium* sp.
PCR Amplicons				
-Accession no.	MT000100	N/A	MT000101	MT000102
-Sequence length	242 bp		244 bp	244 bp
Top BLASTn	~89%	N/A	~88%	~88%
	HM119586.1		HM119586.1	HM119586.1

* Fouling was determined by the presence/absence of epibionts on the carapace.
